# Negative attitudes and lack of Knowledge towards mental health problems

**DOI:** 10.1192/j.eurpsy.2023.2385

**Published:** 2023-07-19

**Authors:** N. Bouattour, F. Cherif, W. Abid, F. Guermazi, R. Massmoudi, S. Hentati, R. Sellami, I. Feki, I. Baati, J. Massmoudi

**Affiliations:** 1 Psychiatry A, Hedi Chaker University Hospital, Sfax, Tunisia

## Abstract

**Introduction:**

Negative thoughts towards mental illness are a global problem for health care professionals. Mainly it leads to late help seeking which aggravates the prognosis of the problem, denial of this situation, refusing long term medication etc.…

**Objectives:**

We aim to identify the determinants leading to negative attitudes towards psychiatric problems among medical students.

**Methods:**

This is a descriptive and analytical cross-sectional study conducted at the Faculty of Medicine of Sfax through an anonymous questionnaire via google Forms. The degree of stigmatization was evaluated by the score « The Attribution Questionnaires AQ-27 ».

**Results:**

One hundred and seven students completed the questionnaires. The Sex-ratio was 0.30 (M/F). The percentage of first- and second-year students was 24.42%, third to 6^th^ year students was 34.57% and residents was 41.01%. Students with a personal history of psychiatric disorders presented 45.8% of our population and those with a family history of mental health problems 40.2%. Medical students who studied psychiatry as a discipline and students who had internship in the psychiatry department (third to 6^th^ year students and residents) had lower scores of the Attribution Questionnaires AQ-27 (p=0,003 and p=0,002 respectively). Sixty per cent of the students reported that spreading listening cells when needed, media coverage of mental illness and campaigns of awareness can help us reduce mental disease’s related stigma.

**Conclusions:**

To conclude, in order to lower rates of stigmatization of mentally sick people, spreading awareness among medical students can be an important tool in order to understand this situation and to provide a better health care.

**Disclosure of Interest:**

None Declared

